# Pervasive effects of RNA degradation on Nanopore direct RNA sequencing

**DOI:** 10.1093/nargab/lqad060

**Published:** 2023-06-09

**Authors:** Yair D J Prawer, Josie Gleeson, Ricardo De Paoli-Iseppi, Michael B Clark

**Affiliations:** Centre for Stem Cell Systems, Department of Anatomy and Physiology, The University of Melbourne, Parkville, VIC, 3010, Australia; Centre for Stem Cell Systems, Department of Anatomy and Physiology, The University of Melbourne, Parkville, VIC, 3010, Australia; Centre for Stem Cell Systems, Department of Anatomy and Physiology, The University of Melbourne, Parkville, VIC, 3010, Australia; Centre for Stem Cell Systems, Department of Anatomy and Physiology, The University of Melbourne, Parkville, VIC, 3010, Australia

## Abstract

Oxford Nanopore direct RNA sequencing (DRS) is capable of sequencing complete RNA molecules and accurately measuring gene and isoform expression. However, as DRS is designed to profile intact RNA, expression quantification may be more heavily dependent upon RNA integrity than alternative RNA sequencing methodologies. It is currently unclear how RNA degradation impacts DRS or whether it can be corrected for. To assess the impact of RNA integrity on DRS, we performed a degradation time series using SH-SY5Y neuroblastoma cells. Our results demonstrate that degradation is a significant and pervasive factor that can bias DRS measurements, including a reduction in library complexity resulting in an overrepresentation of short genes and isoforms. Degradation also biases differential expression analyses; however, we find that explicit correction can almost fully recover meaningful biological signal. In addition, DRS provided less biased profiling of partially degraded samples than Nanopore PCR-cDNA sequencing. Overall, we find that samples with RNA integrity number (RIN) > 9.5 can be treated as undegraded and samples with RIN > 7 can be utilized for DRS with appropriate correction. These results establish the suitability of DRS for a wide range of samples, including partially degraded *in vivo* clinical and post-mortem samples, while limiting the confounding effect of degradation on expression quantification.

## INTRODUCTION

Cellular fate and function are tightly regulated by networks of expressed genes. Therefore, accurate quantification of the transcriptome is crucial in understanding how cells behave in physiological and disease contexts. RNA sequencing (RNA-seq) has become the prevailing method for quantifying the abundance of RNAs and elucidating the complex and dynamic nature of the transcriptome (1–3).

Numerous factors affect RNA abundance, including the rates of transcript production and decay. RNA degradation has a substantive impact on the transcriptome and can occur both through regulated processes within living cells to remove unwanted RNAs and due to cellular damage and death (4–12). Regulated decay rates for individual RNAs can vary 100-fold or more (13) and are affected by a variety of cellular stimuli and mRNA features such as %GC (14), translational termination codon position and AU-rich elements (12,15). Death of the organism or removal of cells from their tissue environment can also lead to mRNA degradation due to ischaemia and eventual cell death, while sample handling and storage practices such as freeze–thawing can compromise cellular membrane integrity, exposing mRNA to RNases (4,12).

Appropriately controlling for RNA degradation when assessing gene and isoform expression levels can be challenging and degradation is often unavoidable when working with post-mortem, clinical or field samples. Such samples may have undergone both regulated and stochastic RNA degradation caused by factors such as storage, handling and necrosis. Therefore, RNA expression levels may no longer faithfully represent true *in vivo* levels. Profiling samples with low or variable RNA quality requires accounting for the confounding effects of RNA quality on gene and isoform quantification (7).

The standard metric used to assess RNA quality is the RNA integrity number (RIN). RIN is a global measure of RNA quality (on a scale of 0–10) defined by the ratio of 28S to 18S ribosomal RNA (16). More recently, gene/isoform-specific integrity scores, calculated from the RNA-seq datasets under investigation, such as mRIN (17), transcript integrity number (18) and DegNorm (8), have also been developed. These metrics have been used in different ways to account for the effects of degradation on RNA expression quantification. One approach is to exclude samples below an arbitrary RIN cut-off. While simple, this method is not always applicable as it may exclude useable samples and does not account for variability in the RNA integrity of included samples. An alternative approach is to sequence samples and attempt to model and correct for RNA degradation. Such methods assume that transcripts degrade at variable rates for a given level of RNA degradation and implement a model that incorporates gene-specific degradation to account for confounding effects (6–8,19). Studies have found that RIN performs well in accurately assessing mRNA quality and correcting for RNA degradation (7), although it cannot fully account for gene-specific decay rates (6,9), while gene/isoform-specific scores produce mixed results (8).

Recently, third-generation long-read sequencing technologies such as Oxford Nanopore Technologies (ONT) and PacBio have grown in popularity for expression profiling. Long-read methods can sequence entire mRNAs in a single read, enabling improved identification and quantification of genes and isoforms (20). However, long-read methods are potentially more sensitive to RNA degradation as they often attempt to profile only undegraded, full-length mRNAs and/or can be limited to RNAs that retain a polyA tail. In contrast, the RNA fragmentation and random priming of cDNA synthesis in Illumina short-read sequencing allow the detection of incomplete mRNA fragments and can therefore generate useable results from significantly degraded RNA (7,21).

A recently developed long-read sequencing method from ONT allows RNA to be sequenced directly (22). Unlike most long-read methods that require reverse transcription and/or PCR, direct RNA sequencing (DRS) utilizes native RNA, allowing it to profile RNAs as they exist in the cell and reduce potential sequencing biases. DRS can detect novel isoforms (23–27), accurately quantify genes and isoforms (28), characterize RNA modifications (29,30) and measure polyA tails (24,26). However, DRS still requires ligation of a sequencing adaptor to the 3′ end of the RNA, with the default strategy being to ligate the adapter to an intact polyA tail (31). Thus, DRS will only sequence isoforms with an undegraded 3′ end unless alternative strategies such as *in vitro* polyadenylation or sequence-specific adaptors are utilized (32,33). The need for an adaptable 3′ end may therefore introduce degradation biases that are more severe than other sequencing methodologies, potentially making DRS impractical at certain RIN values. It is currently unknown how degradation affects DRS and whether methods used to account for degradation can be implemented to obtain accurate quantification data. Consequently, there is a need to determine a suitable range of RIN values in which we can accurately apply DRS while limiting the confounding effect of degradation on downstream analyses.

To evaluate the impact of RNA degradation on DRS, we created an 8-h degradation time series of human SH-SY5Y cells. We included a range of samples with differing RINs, from a minimum of 7 to a maximum of 10. We found that RNA degradation had a pervasive impact on DRS results, leading to fewer full-length reads, reduced library complexity and an overrepresentation of shorter genes and isoforms. However, almost all genes and isoforms could still be detected in degraded samples and explicit correction for RNA integrity (using RIN) almost completely removed the impact of degradation on differential expression (DE) analyses. Comparison to ONT PCR-cDNA sequencing demonstrated that DRS is not overtly more sensitive to RNA degradation, while DRS of human post-mortem brain confirmed that useable results can be obtained from partially degraded *in vivo* samples. We establish the effects and corrective measures of degradation on DRS and provide a range of RIN values within which reliable data can be generated.

## MATERIALS AND METHODS

### Cell culture

Human SH-SY5Y cells were cultured in growth media under standard conditions (5% CO_2_, 37°C) with DMEM + GlutaMAX (Thermo Fisher, 10567-014), supplemented with 10% foetal bovine serum (Thermo Fisher, A4766801) and 1% penicillin–streptomycin (10 000 U/ml) (Thermo Fisher, 15140122).

### RNA degradation and extraction

To assess the reliability and scope of long-read sequencing on degraded or suboptimal RNA, a degradation time series was performed using the human SH-SY5Y neuroblastoma cell line. SH-SY5Y cells were grown to 100% confluency in T175 flasks. Cells were trypsinized using TrypLE Express Enzyme (Thermo Fisher, 12604-021) and combined into one 50 ml falcon tube, spun gently at 800 rpm to form a cell pellet and supernatant was removed. Two additional wash steps in DPBS (Thermo Fisher, 14190-144) solution removed traces of culture media, TrypLE and cell-free RNA without disturbing the cell pellet. The clean cell pellet was then resuspended by gentle pipetting in 250 μl of DPBS. Two 40 μl T0 control/non-degraded sample aliquots were then collected and placed into 1.5 ml Eppendorf tubes, 1 ml of QIAzol (QIAGEN) was added and pipette mixed to disrupt the cells, and samples were incubated for 5 min and placed on dry ice.

Next, we implemented our degradation method, which we optimized to degrade RNA to a RIN range between 7 and 10. This range retains enough long RNAs to make DRS worthwhile, while at the same time provides a broad enough range to measure the effects of degradation and provides a framework to compare ideal samples (RINs between 9.5 and 10) with high-quality samples that have experienced some RNA degradation (RINs of 7–9).

To degrade RNA in the remaining cells, cells were freeze–thawed by placing the 50 ml falcon tube on dry ice for 10 min before returning it to room temperature. Duplicate 40 μl samples were collected at the following time intervals: 0.5, 1, 2, 3, 4, 6 and 8 h. The cell suspension was gently agitated before sample collection to ensure that sampling was from a homogeneous cell solution. Immediately after collection, cells were disrupted using 1 ml of QIAzol and incubated for 5 min before being placed on dry ice. RNA extractions were performed using RNeasy Lipid Tissue Kit (QIAGEN, 74804) according to the manufacturer’s instructions. A maximum of six samples were extracted at any one time and samples were randomly assigned to extractions to reduce extraction times and minimize batch effects. RNA quality (RIN) was assessed on a TapeStation 4200 (Agilent) and RNA purity on a NanoDrop (Thermo Fisher), while RNA concentration was assessed using a Qubit fluorometer (Thermo Fisher). QC data are available in Supplementary Table S1. The degradation time series was performed on three separate occasions to ensure that replicates were available for each time point. We selected 15 samples for DRS, with at least 2 samples included from each of the six time points. Samples were chosen based on RNA purity and yield. All selected samples had highly pure RNA with low levels of contaminants that could affect DRS library preparation. In addition, they had at least 15 μg of total RNA, enough for polyA+ purification and DRS library construction.

### Library preparation and sequencing

To prepare RNA for sequencing, a minimum of 15 μg of total RNA from each sample was polyA+ purified using 50 ml of NEXTflex polyA+ beads (Bioo Scientific). Samples were randomized, purified and sequenced in groups of 3–5 to reduce batch effects. The concentration of purified RNA was assessed using a Qubit fluorometer. Sequencing libraries were prepared immediately thereafter using the SQK-RNA002 kit (ONT) using a minimum input of 120 ng of polyA+ RNA (Supplementary Table S1). Synthetic sequin V3 spike-in RNA controls (34) were added to each sample at 10% of total mRNA, with control samples receiving Mix A and all other samples Mix B. Libraries were sequenced on the GridION (ONT) using FLO106 flow cells and MinKNOW (v20.10.6) to generate FAST5 files. FAST5 files were basecalled with Guppy (v3.5.2) (ONT) to create summary text files and FASTQ files.

RNA from 250 mg of post-mortem cerebellum tissue (obtained from the Victorian Brain Bank) was extracted and polyA+ purified according to the methods stated above. SQK-RNA002 library preparation was performed with 500 ng of polyA RNA as input and sequenced on a PromethION flow cell (FLO-PRO002). FAST5 files were basecalled with Guppy (v3.5.2) (ONT). Nanopore PCR-cDNA sequencing was also performed on four samples, two control (T0) and two 6 h post-degradation treatment, using the SQK-PCS110 kit (ONT) as per the manufacturer’s instructions. Sequin controls (34) were included at 10% as per dRNA sequencing. cDNA samples were sequenced on the GridION using FLO106 flow cells using MinKNOW (v20.10.6) and basecalled with Guppy (v4.2.3) (ONT).

### Sequencing metrics and quality control

Initial data analysis was performed using pycoQC (35) to gather general sequencing metrics. All analyses were performed on pass reads [quality (*Q*) score ≥7] unless otherwise stated.

### Genome and transcriptome alignment

FASTQ files containing ONT pass reads were aligned to the human (GRCh38) (36) and synthetic sequin (34) genome and transcriptome using minimap2 (v2.17) (37). The genome alignments were performed using the splice-aware mode of minimap2 *-ax splice -uf* as recommended. The transcriptome alignments were performed using the long-read mode for ONT data *-ax map-ont -k14* to retain multiple secondary alignments.

### Full-length transcript identification

Read and isoform coverage fractions were calculated and full-length transcripts identified from transcriptome BAM files using BamSlam (https://github.com/josiegleeson/BamSlam) (28). Read coverage fractions were used to assess the proportion of original transcript length covered by each mapped read. Read coverage fractions were calculated by dividing the length of each read’s best alignment (alignment with highest alignment score) by the known isoform length. We also calculated an isoform coverage fraction, defined as the median read coverage fraction per detected isoform. Reads were required to cover at least 95% of their annotated isoform to be classed as full length. As a complementary metric for full-length reads, we also calculated the percentage of reads that aligned within 50 nt of the annotated 3′ end and within 25 nt of the annotated 5′ end of their assigned isoform as per Workman *et al.* (26). Gene body coverages were calculated using RSeQC (38) using standard parameters.

### Filtering for single isoform genes

We filtered the comprehensive gencode.v31.gtf file for single isoform genes. We extracted gene IDs and a bed file containing genomic coordinates for all single isoform genes using the following script: https://github.com/Sefi196/RNA_Deg/blob/main/scripts/filter_gtf_for_single_isoform_genes.R. Next, we filtered the BamSlam output files using the single isoform gene list and then generated isoform coverage plots. To generate gene body coverages, we filtered bam files with bedtools v2.3 (39) using the intersect feature. We filtered for reads that are contained within the single isoform gene bed file. Then, gene body coverages were calculated using RSeQC (38) using the filtered bam files. The complete workflow is available at https://github.com/Sefi196/RNA_Deg/blob/main/scripts/single_isoform_gene_body_coverage.sh.

### Gene and isoform quantification and differential expression

Quantification at the gene level was performed using featureCounts (v1.6.5) (40). Isoform quantification was performed with Salmon (v1.10.1) (41) and NanoCount (v1.0.0) (28). The commands used for each quantification tool can be found at https://github.com/Sefi196/RNA_Deg/blob/main/scripts/Quantification_commands.sh. Both human and sequin transcripts were quantified and estimated counts were used for DE analysis. DE of genes and isoforms was detected using the R package DESeq2 (v1.32.0) (42) utilizing a GLM framework with time to lysis as a factor (∼time), and batch effects and sample RIN as covariates. Only genes or isoforms with a minimum of 10 normalized counts in at least five samples were considered for DE analysis. The full DE workflow can be found at https://github.com/Sefi196/RNA_Deg/blob/main/scripts/differentialExpression-workflow.R. To investigate the impacts of degradation on quantification, we extracted TPM values from NanoCount and Salmon and plotted the TPM of the control samples (T0) against all other time points (T1–T5). Next, we fit a linear regression and calculated Pearson’s and Spearman’s correlation coefficients. Quantification was performed with the same input bam files for both NanoCount and Salmon.

### Genes and isoforms that can no longer be detected

To identify genes and isoforms that could no longer be detected at 8 h post-freeze–thaw treatment, we used normalized gene and isoform counts obtained from the DESeq2 pipeline. Undetectable genes and isoforms were defined as per the following criteria:

1. Five or more normalized counts in at least four of the first five samples (T0 and T1).
2. Zero normalized counts in both samples at 8 h.
3. We fit an exponential decay curve to each gene or isoform and selected those with a Pearson’s correlation coefficient (*r*) ≥ 0.5.

### *K*-means clustering and gene enrichment

Log fold change values produced by DESeq2 were used for gene and isoform clustering. Clustering was performed with the stats package (v4.1.0) in R implementing a *K*-means clustering algorithm with 100 iterations. Genes and isoforms were clustered into four groups representing the following: (i) apparent upregulation (upregulation and increased relative abundance of highly stable RNAs); (ii) stable (i.e. that maintain or slightly increase in apparent abundance); (iii) slow degradation; and (iv) fast degradation. Log fold change values were normalized using the centroid values from cluster 2. This normalization accounts for relative changes in abundance that may make stable genes appear upregulated as genes or isoforms degrade throughout the time series. To compare gene/isoform architecture between the degradation clusters, we extracted gene and isoform feature information (UTR length, gene length and CDS length) from the comprehensive GENCODE v31 annotation file. We extracted %GC content from the BioMart database v2.0 (43). The full workflow is available at https://github.com/Sefi196/RNA_Deg/blob/main/scripts/Gene_Isoform_architecture.R. Statistical comparisons between clusters were performed with an ANOVA and post-hoc analysis using Tukey’s test. Identification of overrepresented Gene Ontology (GO) terms among each cluster was performed with Goseq (v1.44.0) (44) applying the ‘hypergeometric*’* method. The background dataset used in all cases included the 7864 genes that passed filtering steps and was used for DE and *K*-means clustering.

## RESULTS

### Degradation time series to model changes in RNA quality

**Figure 1.**
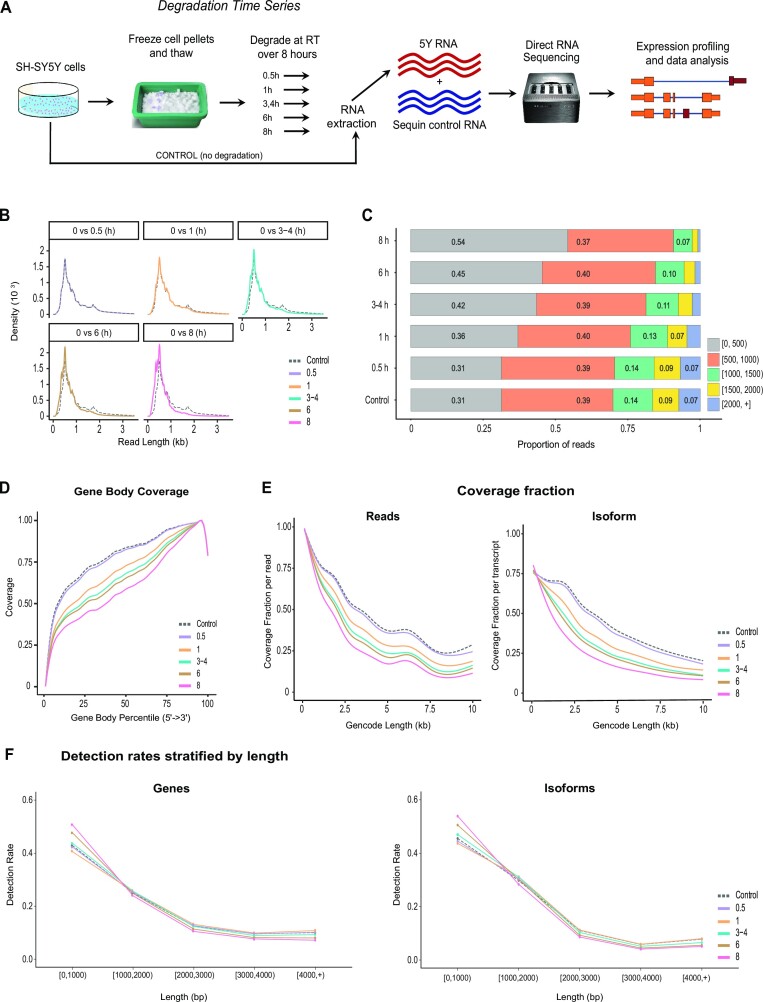
Experimental overview and DRS degradation metrics. (**A**) SH-SY5Y cells were cultured, freeze–thawed and left at room temperature for up to 8 h. Native polyA purified SH-SY5Y RNA was combined with ‘sequin’ spike-in RNA and prepared for DRS on an Oxford Nanopore GridION. Reads were analysed to identify the effect of degradation on library sequencing metrics and differential gene and isoform analysis. (**B**) DRS read length distributions. Control read length distribution shown as a dashed line compared to all other time points. (**C**) Proportion of reads from each time point stratified by read lengths. The bar graph indicates the proportion of reads within 500 nt bins. Numbers on bars represent the proportion of reads in that bin rounded to the nearest whole number. Segments representing <5% are not labelled. (**D**) Gene body coverage of SH-SY5Y reads. Length of all genes has been normalized to 100 and plotted from 5′ (0) to 3′ (100). Lines show mean coverage for all genes across the gene body length. Lower coverage at extreme 3′ corresponds to soft clipping of the first bases sequenced that often have lower phred quality. (**E**) Read and isoform coverage fractions. Read coverage fraction (left): fraction of isoform length covered by each read. Isoform coverage fraction (right): median coverage fraction of all reads mapped to each isoform. *X*-axis range limited to 10 kb as majority of reads and isoforms lie within this range. (**F**) Detection rates stratified by length. Proportion of genes and isoforms detected (count ≥1) stratified by length. Samples with RIN < 9 show higher detection rates at short lengths (<1000 bp) and lower detection rates at long lengths (>1000 bp) compared to control.

As RNA from post-mortem, clinical or field samples is often partially degraded prior to sequencing, we set out to examine the effects of this degradation on long-read DRS. We implemented a degradation time series on SH-SY5Y neuroblastoma samples (Figure 1A) (see the ‘Materials and Methods’ section) where samples were freeze–thawed in PBS and then left at room temperature for up to 8 h. This treatment replicates aspects of *in vivo* samples, including loss of external nutrients, cellular stress and loss of membrane integrity. Three replicates of the degradation time series were performed. We selected 15 samples for DRS from six time points [0 (control), 0.5, 1, 3 or 4, 6 and 8 h), with at least 2 sample replicates included at each time point.

Sample RINs ranged from 9.9 for the 0 h controls to 7.2 at 8 h (Table 1 and Supplementary Table S1). We hypothesized that DRS would be highly sensitive to RNA degradation and so targeted RINs between 7 and 10, as this is a plausible range for many higher quality post-mortem, clinical or field samples. Furthermore, to investigate the possibility that even small shifts in RNA integrity might affect DRS results, we created a time series with only small (<1) changes in RIN between each time point.

**Table 1.** DRS metrics

Time post-degradation treatment (h)	Average RIN	N50 (bp)	Median aligned length (bp)	Full-length transcripts (%)	Transcripts within 25 bp of 5′ and 50 bp of 3′ (%)	Median coverage fraction per read	Median coverage fraction per isoform	Number of genes detected per 1M reads	Number of isoforms detected per 1M reads
0 (*n* = 3)	9.8	1378	773	25	20	0.68	0.53	14 727	33 502
0.5 (*n* = 2)	9.6	1460	774	24	19	0.67	0.52	14 849	34 387
1 (*n* = 2)	9.3	1203	697	20	17	0.57	0.42	14 942	34 431
3–4 (*n* = 3)	8.7	1009	628	19	17	0.53	0.38	14 733	35 164
6 (*n* = 3)	8.2	925.6	607	20	18	0.53	0.35	14 663	34 043
8 (*n* = 2)	7.25	777	518	16	16	0.47	0.29	14 459	33 806

### Sample degradation impedes full-length isoform detection and introduces a short isoform bias

As expected, a strong, negative correlation was identified between time to lysis and RNA integrity, *r* = −0.95 and *P* < 0.001 (see Supplementary Table S1). Sample RIN was also strongly associated with key sequencing metrics, including sample N50, *r* = 0.78 and *P* < 0.001; median aligned length (primary alignments only), *r* = 0.83 and *P* < 0.001; percentage of full-length transcripts, *r* = 0.68 and *P* < 0.001; and median coverage fraction at both the read, *r* = 0.76 and *P* < 0.001, and transcript isoform levels, *r* = 0.84 and *P* < 0.001 (Table 1). Throughout the time series, we observed reductions of 44% in read N50, 33% in median aligned read length, 36% in full-length transcripts, and 31% and 45% in read and isoform coverage fractions, respectively (Table 1). An alternative definition of full-length transcripts showed a similar trend, with a 20% reduction in reads aligned within 25 bp of the 5′ and 50 bp of the 3′ of their assigned isoforms. These demonstrate that changes between RINs 7 and 10 represent large changes to the underlying mRNA. No significant changes in global sequencing metrics were observed between 0 (control) and 0.5 h samples, suggesting that changes in samples with RIN > 9.5 are below the detection threshold of DRS and/or that small differences in samples with RINs above 9.5 do not represent meaningful differences in RNA integrity.

To further assess the impact of degradation-induced changes on overall read lengths, we compared read length distributions at baseline with every other time point (Figure 1B and C). Changes in read lengths are minimal at 0.5 h; however, poorer quality samples contain an increasing proportion of shorter read lengths. Our data confirm that longer reads become increasingly difficult to sequence below a RIN of ∼8.5, with <5% of reads exceeding a length of 1.5 kb compared to 16% in the baseline control samples (Figure 1C).

Next, we examined read coverage across gene bodies (Figure 1D). As DRS requires intact 3′ ends, which are sequenced first, we observe a 3′ bias in coverage, consistent with previous reports (28,31,45). RNA degradation exacerbates this 3′ bias, with substantial declines in gene body coverage below a RIN of 9.5. We then asked whether a decrease in coverage depended on isoform length. In undegraded control samples, both read and isoform coverage fractions show similar trends, where increasing isoform length correlated with a decrease in coverage, as shown previously (23,28) (Figure 1E). This likely represents increased sensitivity of longer RNAs to degradation during RNA extraction and library preparation. The same trend is evident and becomes progressively more prominent as samples degrade. The effect is not uniform, however, as short isoforms (<600 bp) exhibit very similar coverage fractions to those in undegraded samples, while longer isoforms (>600 bp) display a greater rate of coverage fraction decline (Figure 1E and Supplementary Figure S1A and B). The rate of coverage fraction decline is significantly correlated with RIN (read coverage: *r* = 0.76 and *P* < 0.001; isoform coverage: *r* = 0.84 and *P* < 0.001) and has the largest impact on isoforms between 750 and 2000 bp long (Figure 1C). These results show that longer isoforms are more significantly affected by RNA degradation, rendering detection of long full-length isoforms more difficult with lower quality samples. Conversely, detection of short full-length transcripts appears to be minimally impacted by degradation, demonstrating that lower quality samples may still be suitable when examining expression of short genes and isoforms.

We next asked whether RNA degradation affected gene and isoform detection rates. While RNA degradation did not significantly impact the number of detected genes and isoforms (*P* = 0.98 and *P* = 0.82, Supplementary Figure S2), a higher proportion were under 1 kb in lower RIN samples, with a corresponding decrease in the proportion above 1 kb (Figure 1F). Collectively, these data substantiate that library complexity is impacted by degradation, with longer features being more susceptible to degradation, while shorter genes and isoforms remain more resistant to degradation resulting in a short gene and isoform bias.

To verify that the short gene and isoform bias we detected is a result of degradation and not a consequence of incorrect isoform assignment, we performed the same analysis on single isoform genes. By doing so, we removed the possibility of incorrect isoform assignment, which could result in overestimation of some transcripts over others. Our results show that degradation has the same effect on single and multiple isoform genes (Supplementary Figure S1C and D) indicating that isoform estimation error is not a major factor impacting these findings.

### Assigning reads to isoforms becomes more challenging as samples degrade

**Figure 2.**
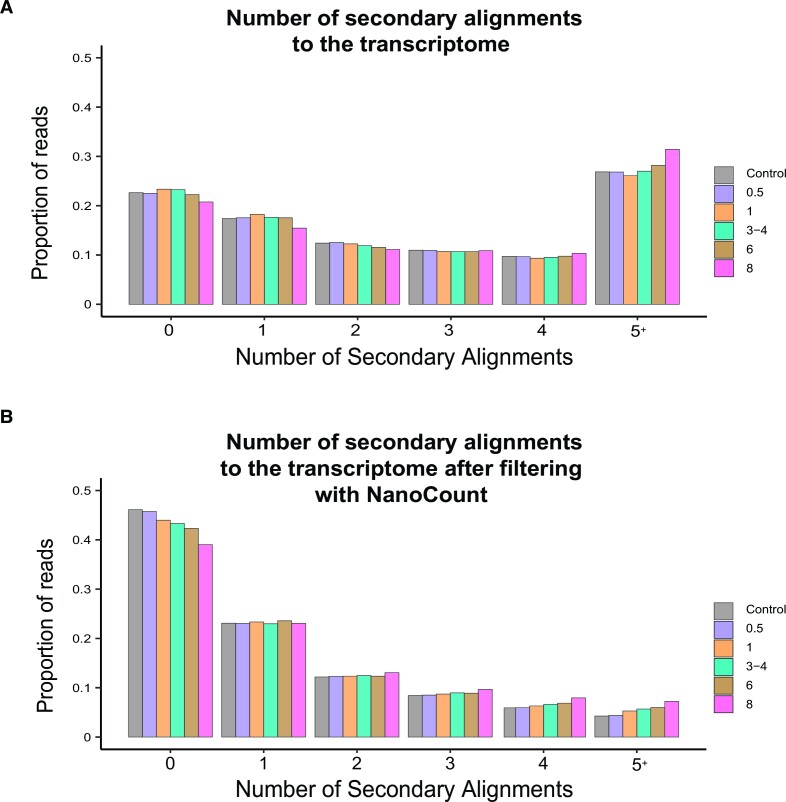
The proportion of reads with secondary alignments to the transcriptome: (**A**) with minimap2 and (**B**) after filtering with NanoCount. NanoCount was used to filter out alignments with a 3′ >50 nt away from an annotated transcript end and an alignment score <95% of the best alignment score for that read.

The human transcriptome contains many highly similar isoforms, which leads to difficulties in assigning DRS reads to their correct isoform of origin (31,46). We recently developed the NanoCount software package to improve DRS read assignment and quantification (28). After filtering our data using NanoCount, we find a substantial improvement in general sequencing metrics (Figure 2A and B, and Supplementary Table S2), including an increased proportion of reads with only a primary alignment (∼20%).

Given the important role of full-length reads in accurate isoform assignment, we hypothesized that assignment would be more ambiguous as samples degrade, leading to more reads with multiple (i.e. secondary) alignments. Supporting this, we observed a correlation between the proportion of reads with five or more secondary alignments and decreased RIN (*r* = 0.90 and *P* < 0.001, Figure 2B). However, the impact of decreased RIN was small compared to the improvement obtained with NanoCount, suggesting that NanoCount performs well on partially degraded RNA and that accurate quantification of isoforms should be feasible.

### Almost all genes and isoforms are still detectable in degraded samples

We wanted to determine whether there were any genes and isoforms we could no longer detect at 8 h post-degradation treatment. Nearly all genes and isoforms with detectable expression at T0 and/or 0.5 h were detected throughout the entire time series, with only 2 genes (Supplementary Figure S3A) and 34 isoforms (Supplementary Figure S3B) no longer detectable at 8 h. A majority (68%) of these isoforms showed classical exponential decay patterns with Pearson’s correlation coefficient *r* ≥ 0.7, supporting their susceptibility to degradation. However, both the genes and 82% of the isoforms were lowly expressed (<25 normalized counts) at T0, demonstrating that genes and isoforms that are no longer detectable at 8 h are likely to be those that are lowly expressed to begin with and therefore fall below the detection threshold for DRS during the time series. Almost all genes and isoforms can still be detected at a RIN of ∼7.

### Degradation rates are associated with gene and isoform architecture

**Figure 3.**
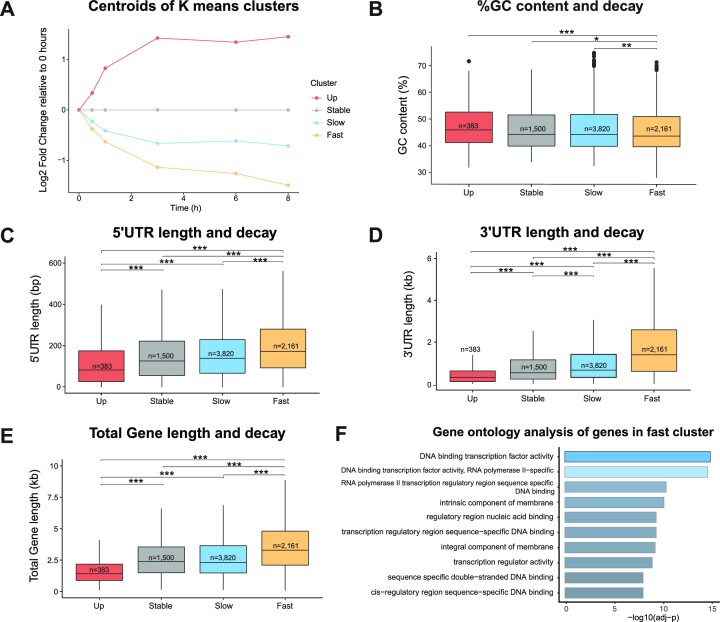
Characteristics of gene degradation clusters. Up, upregulated; slow, slow degradation; fast, rapid degradation. (**A**) Centroid of each *K*-means cluster derived from log_2_ fold change data scaled to 0 h. %GC (**B**), 5′ UTR length (**C**), 3′ UTR length (**D**) and complete transcript length (UTRs + CDS) (**E**) of genes in each cluster. In the interest of clarity, panels (C)–(E) omit outliers (1.5 × IQR). Outliers are included in statistical analysis. All lengths (UTRs and total gene lengths) are median values. *P < 0.05, **P < 0.01 and ***P < 0.001. Statistical comparisons between clusters were performed with an ANOVA and post-hoc analysis using Tukey’s test. (**F**) GO terms most associated with genes in the fast-degrading cluster. The 10 most significant terms are shown. *P*-values adjusted using Bonferroni correction for multiple testing.

We sought to increase our understanding of gene and transcript degradation dynamics in our time series. Using *K*-means clustering, we grouped log fold change data (relative to 0 h) into four groups (each group outlining how gene/isoform abundances change over time): (i) Genes or isoforms that have apparent upregulation. This may seem counterintuitive given that most changes in abundance are likely the result of RNA degradation; nevertheless, as our experimental design treats cells and does not inhibit transcription, it is possible for some cells to be transcriptionally active throughout the time series. (ii) Genes or isoforms that are stable (i.e. that maintain or slightly increase in apparent abundance). (iii) Genes or isoforms that degrade slowly. (iv) Genes or isoforms that degrade rapidly. Of the 7864 genes clustered, we identified 4.9% (383) that show apparent upregulation, 19.1% (1500) that are stable, 48.6% (3820) with slow degradation and 27.5% (2161) with rapid degradation (Figure 3A and Supplementary Figure S4).

Previous short-read studies have identified RNA characteristics that associate with degradation rate (7). To investigate whether similar results are obtained with DRS, we looked for gene characteristics that were associated with degradation clusters. By ANOVA we found significant differences between clusters in GC content (*P* < 0.001), UTR lengths (5′ and 3′) (*P* < 0.001), CDS lengths (*P* < 0.001) and total gene length (5′ UTR + CDS + 3′ UTR) (*P* < 0.001) (Figure 3). Genes that degrade faster have lower GC content (0.6%) (Figure 3B) and longer UTRs (5′ UTR 48 bp and 3′ UTR 932 bp) (Figure 3C and D), CDS lengths (124 bp) and total gene lengths (947 bp) (Figure 3E) when compared to the stable cluster. We observe similar trends between isoform clusters (Supplementary Figure S5) and in single isoform genes (Supplementary Figure S6), though manual inspection of isoforms in the ‘Up’ cluster (Supplementary Figure S5) suggested that it may be enriched for isoforms whose expression appears to change due to degradation-induced read misassignment. As shown by the Eta-squared values, differences in 3′ UTR lengths were the most important factor separating each cluster (Supplementary Table S3). Since DRS generally requires an intact polyA tail to sequence an RNA, this highlights how 3′ UTR length/stability is the key factor affecting transcript detection and quantification with DRS. Our data thus suggest that the lengths of all gene/isoform features are relevant factors that affect degradation, emphasizing that degradation rates are gene and isoform specific.

We investigated whether degradation clusters differ meaningfully in terms of biological categories (Supplementary Table S4). Among the rapidly degraded cluster, we found a clear overrepresentation of GO terms associated with DNA binding proteins and transcription factors (Figure 3F). These data agree with previous findings, which have also demonstrated that transcription factor mRNAs degrade quickly (47). The upregulated cluster contained 10 molecular chaperones, which could be a response to the cellular stress introduced in our degradation treatment, including *HIF1A*, a gene known to regulate cellular responses to hypoxia (Supplementary Table S5). These GO enrichments further demonstrate the non-random nature of RNA degradation and highlight the importance of accounting for degradation biases in DRS experiments.

### RNA degradation hinders DE analysis

**Figure 4.**
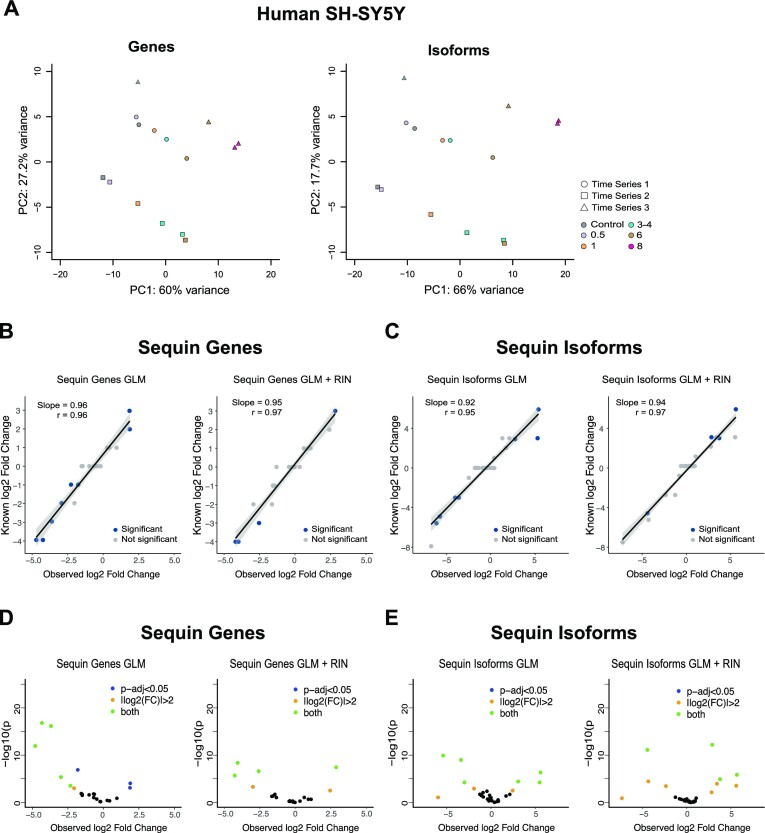
Differential gene and isoform expression. (**A**) PCA of samples grouped by time. PCAs generated with gene and isoform counts obtained using featureCounts and NanoCount, respectively. Colours show RIN/time points and shapes show degradation experiments. (**B**, **C**) Quantification of fold changes between Mix A and Mix B sequin genes and isoforms using a standard GLM or GLM + RIN. Sequins with significant DE are in blue. Trend line shows slope from linear regression with shaded grey region indicating a 95% confidence interval for regression slope. Correlation (*r*) is Spearman’s correlation. (**D**, **E**) Volcano plots of sequin genes and isoforms with/without the inclusion of sample RIN in GLM. Adjusted *P*-value <0.05 was considered significant for DE.

A key goal in transcriptomic studies, including DRS, is identification of DE of genes and isoforms, which requires accurate and sufficiently unbiased expression quantification. Principal component analysis (PCA) confirmed that RNA integrity was the major contributor to sample variance at both the gene and transcript levels (PC1 60% and 66%, respectively) (Figure 4A). PC2 separated samples by degradation replicate, despite randomization of sample processing from RNA extraction through to sequencing, suggesting that RNA degradation is quite heterogeneous, even under identical conditions.

Given the significant effects of RNA degradation on the transcriptome, we investigated what impact this had on differential gene and isoform expression analyses. Genes and isoforms identified as DE should largely be a result of degradation treatment rather than true biological signal. We assessed gene and isoform DE with DESeq2 (42) accounting for replicates (∼replicate effects + time).

We did not detect any DE genes between the 0 h control and 0.5 h post-treatment, further demonstrating that the effects of degradation at this early time point are minimal. At 1 h post-treatment, we detected 36 DE genes. Both the number of DE genes and the magnitude of expression changes increased as RNA degrades reaching 381 DE genes by 8 h, with a similar trend evident at the isoform level (Table 2). Isoform DE was tested following quantification with both Salmon (41) and NanoCount. NanoCount was considerably less susceptible to RNA degradation-induced false-positive DE identification (0 versus 213 at 0.5 h, 549 versus 762 at 8 h).

**Table 2.** DE genes and isoforms

	GLM				GLM + RIN		
Time point	DE genes (featureCounts)	DE genes (DegNorm)	DE isoforms (Salmon)	DE isoforms (NanoCount)	DE genes (featureCounts)	DE isoforms (Salmon)	DE isoforms (NanoCount)
0 versus 0.5 h	0	0	213	0	0	154	0
0 versus 1 h	36	2	265	28	4	35	0
0 versus 3–4 h	164	74	239	207	12	7	10
0 versus 6 h	169	232	314	259	0	0	0
0 versus 8 h	381	439	762	549	0	0	0

Number of genes/isoforms tested for DE: featureCounts, 7885; DegNorm, 9158; Salmon, 11 993; NanoCount, 10 607.

We investigated the impact degradation had on NanoCount and Salmon quantification by comparing isoform counts at 0 h to all other time points (Supplementary Figure S7). Results showed consistently higher correlations with NanoCount and less impact from degradation, demonstrating that quantification with NanoCount is more robust to sample degradation. Thus, the lower number of NanoCount false positives is likely due to improved isoform assignment reducing the impact of degradation on isoform quantification and hence the biases in the GLM model. This is further supported by the number of NanoCount DE isoforms more closely resembling the number of DE genes, where correct assignment is not a major issue. Taken together, these results demonstrate that even modest RNA degradation (RIN 7) can bias expression quantification and leads to a large number of DE false positives if not corrected for.

### Correction of DRS degradation biases on differential expression

Our GLM framework shows clear evidence of a degradation bias as detection of DE genes and isoforms is largely a result of degradation and not a real biological effect. We therefore tried to account for these degradation biases by adding sample RIN as a covariate to our model (∼RIN + replicate effects + time). We also compared our GLM to the DegNorm pipeline (8) for gene DE (Table 2). Including RIN as a covariate drastically reduced the number of DE genes and isoforms across time points (Table 2). Using a GLM + RIN also outperformed DegNorm gene-specific degradation modelling, though DegNorm was designed for short reads and may perform suboptimally on DRS. NanoCount again outperformed Salmon and therefore all downstream isoform analyses were performed with NanoCount. The observation that the inclusion of RIN drastically reduced the number of DE genes and isoforms not only shows that sample RIN is an accurate measure of sample degradation, but also demonstrates that the addition of RIN into a GLM framework is sufficient in reducing degradation-induced variation to almost zero for DRS.

### The addition of RIN in the GLM does not bias DE analysis

To confirm that RIN does not overcorrect our model and mask true expression changes between time points, we used synthetic controls (sequins) to assess the impact of RIN on the GLM. We added synthetic mixes into our samples prior to library preparation after the degradation time series; therefore, sequins should not show degradation over time. Control (*t* = 0) samples received Mix A, while all other samples received Mix B. Mix A and Mix B contain the same synthetic genes and isoforms in different concentrations to simulate changes in expression. Visualizing the sequin data using a PCA demonstrated separation of Mix A and Mix B samples (Supplementary Figure S8).

Our results confirm that concentration changes in sequin genes and isoforms were accurately measured in both the GLM and GLM + RIN analyses, with the correlations between the known and observed sequin fold changes all ≥0.95 with slopes close to 1 (Figure 4B and C). Furthermore, we detected true DE of sequin genes and isoforms after inclusion of RIN and no false-positive DE. These results suggest that correcting for RIN does not overcorrect the data to abolish DE detection or create false-positive results. However, inclusion of RIN does increase the number of DE false negatives (Figure 4D and E), likely due to the inclusion of additional covariates in the model reducing experimental power. Increased sequencing depth would help overcome this issue as sequin genes and isoforms with relatively large counts and log fold changes (absolute log fold change >2.5) are detected as DE using either framework. Overall, our results confirm that the inclusion of RIN does not prevent detection of true DE by DRS while removing almost all degradation-induced false positives.

### Comparing degradation due to freeze–thaw with post-mortem tissue

We wanted to confirm that degradation induced by freeze–thaw was comparable to a real biological sample that had undergone degradation due to normal handling and processing. We performed DRS on a post-mortem human brain sample with RIN = 7.8 and compared sequencing metrics to samples from our time-series data. The percentage of full-length transcripts detected, as well as median coverage fractions at the read, isoform and gene body levels, falls within the range of a high-quality but partially degraded sample (Table 3 and Supplementary Figure S9). Both the N50 and median aligned lengths were longer than the SH-SY5Y samples, which is likely a consequence of differences in gene and isoform expression between SH-SY5Y cells and cerebellum. These data show that RNA degradation induced by freeze–thaw is a useful model for the effects of degradation in decaying or dying tissue.

**Table 3.** Comparing PCR-cDNA and DRS sequencing metrics

	Time post-degradation treatment (h)	Average RIN	N50 (bp)	Median aligned length (bp)	Full-length transcripts (%)	Median coverage fraction per read	Median coverage fraction per isoform
Brain		7.8	1730	904	21	0.52	0.44
PCR-cDNA	0 (*n* = 2)	9.8	1230	575	38	0.84	0.51
	6 (*n* = 2)	7.95	879	498	29	0.62	0.33
DRS	0 (*n* = 2)	9.8	1527	778	26	0.70	0.55
	6 (*n* = 2)	7.95	957	623	20	0.54	0.36

### Comparison of long-read PCR-cDNA and DRS sequencing of degraded samples

**Figure 5.**
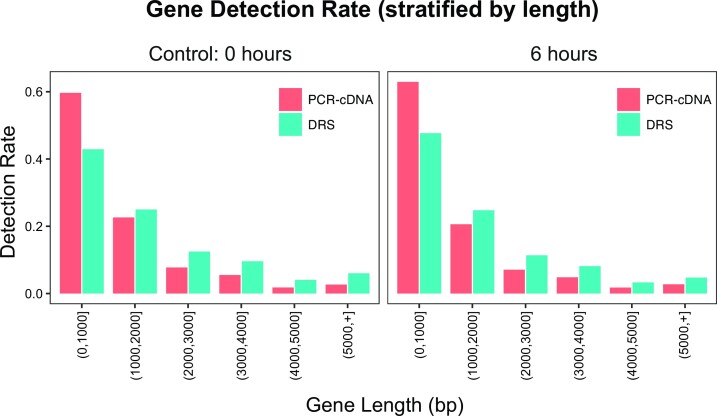
Comparison of detection rates for PCR-cDNA and DRS sequencing at 0 and 6 h. Gene detection rates are stratified by 1 kb gene length intervals. PCR-cDNA sequencing detects a higher proportion of shorter genes, while DRS detects a greater proportion of longer genes.

In addition to DRS, we generated data using Oxford Nanopore PCR-cDNA sequencing on four SH-SY5Y samples (two at 0 h and two at 6 h). We were interested in comparing these two sequencing approaches to assess which would be most suitable for profiling partially degraded RNA. The PCR-cDNA protocol should enrich for full-length transcripts, and we hypothesized that this would make it less susceptible to RNA degradation. Our results show that PCR-cDNA produced a greater percentage of full-length transcripts as expected, but that read lengths were shorter for both intact and partially degraded RNA (Table 3). Stratifying by length demonstrated that PCR-cDNA libraries were biased towards genes and isoforms under 1 kb and had a lower detection rate of longer genes and isoforms (Figure 5). These findings are likely a result of the PCR step biasing read lengths, as shorter molecules amplify more efficiently. In the partially degraded samples, we see less of a difference between sequencing methods (Table 3); however, DRS still provided a less biased representation of the transcriptome when compared to the PCR-cDNA samples at 6 h.

## DISCUSSION

DRS offers the unique ability to measure RNA as it exists in the cell, including sequencing complete RNA molecules and accurate quantification of gene and isoform expression levels (28). As applications for DRS expand, it will be crucial to understand the different sample types DRS can be applied to and which analysis methods will ensure accurate results. As many post-mortem, clinical and field samples have some level of RNA degradation, it is important to know how RNA decay impacts DRS and whether these effects can be corrected for and to establish the minimum RNA quality required for DRS. We find that even moderate degradation in what would generally be considered high-quality samples (RIN > 7) has pervasive impacts on mRNA and hence DRS results. However, the impact of degradation on a DE analysis can be effectively corrected for. Our results inform the use of DRS by providing a framework for analysing partially degraded samples, or a range of samples with differing RINs. DRS can therefore be applied to a variety of samples with varying quality while limiting the confounding effect of degradation on downstream analysis.

Our time series of RNA degradation focused on samples with a RIN between 7 and 10. We chose this range because clinical and/or post-mortem samples commonly fall within this range and we hypothesized that long-read sequencing of native RNA would be highly sensitive to degradation. As reasonably intact RNA is required to enable many of the advantages of long-read RNA-seq methods, the RIN range tested in this study is smaller than previously examined for short-read RNA-seq where highly degraded RNA (RIN < 4) was also utilized (7). We demonstrate that DRS is susceptible to degradation-induced biases affecting global sequencing metrics, reducing both library complexity and the ability to accurately profile long genes and isoforms. RNA degradation impacts DRS in two broad ways: 5′ or internal degradation that decreases read coverage and isoform assignment and 3′ degradation impacting quantification, especially of genes and isoforms with long 3′ UTRs. A known limitation of DRS is accurate isoform assignment (28,31,46), which is largely attributable to non-full-length reads and the many highly similar isoforms present in complex transcriptomes. The impacts of degradation exacerbate assignment ambiguity due to lower coverage towards the 5′ end of transcripts, increasing the difficulty in distinguishing similar isoforms. Therefore, isoform assignment errors also have the potential to lead to isoform quantification errors and such issues should be carefully considered when dealing with samples of lower quality. The impact of degradation, while pervasive, did not prevent detection of almost all genes and isoforms and the overall decrease in isoform assignment was small. Our data also demonstrated that shorter genes and isoforms remain relatively stable even in suboptimal samples (RIN 7), suggesting that DRS can still be applied without explicit correction if the detection and quantification of short molecules is the primary focus.

DE analyses are central to studies investigating the transcriptome but can be limited by confounding factors. Degradation or sample quality is a factor that can falsely implicate genes and isoforms as DE. Previous studies using short-read RNA-seq have demonstrated the need to correct for sample quality, employing a wide array of correction strategies to minimize these impacts (6–8). We find that the inclusion of RIN as a covariate in our model is sufficient to reduce degradation-induced DE in long reads to almost zero (7). These results suggest that RIN is an accurate measure of sample quality; however, caution is advised as the addition of covariates can reduce sensitivity and may mask true DE genes and isoforms, especially those that have small changes in expression and/or few counts. The fact that degradation effects can be accounted for supports a broad use case for DRS and also shows that DRS will be useful in detecting DE features even in cases with suboptimal RNA.

Previous studies have shown that RIN alone is not sufficient to explain the non-random nature of decaying genes and isoforms (6,9). Although we find RIN is a robust indicator of sample quality, we also find that genes and isoforms decay at differing rates that are closely associated with gene and isoform architecture and function. The nature of DRS could also play a role as degradation can occur from the 5′, 3′ or internally within an RNA (4,12). DRS requires an adaptable 3′ end (most commonly a polyA tail) and therefore differences in transcript susceptibility to different degradation pathways likely impact quantification with DRS irrespective of global RIN. A possible approach to mitigate this issue would be to perform *in vitro* polyadenylation on samples with lower RIN scores. We speculate this would increase 5′ coverage by making mRNAs without intact 3′ ends sequenceable, but would also result in more short reads that may be difficult to assign to isoforms. A combination of polyadenylation and 5′ capture (48) could also help maximize informative and sequenceable reads, but this method remains to be explored.

It is likely that RNA decay differs under different degradation conditions. Degradation of transcripts due to ischaemic cell death likely differs from that induced by freeze–thaw. The former is more closely associated with biologically mediated processes (49), while the latter likely causes ruptured cell membranes and leakage of RNases. As such, freeze–thaw-mediated degradation employed in this study may be more severe as the cellular environment is likely deteriorating at a faster rate compared to that of dying tissue. This may explain the better than anticipated (given the RIN) sequencing metrics obtained from the post-mortem human brain sample and suggests that DRS should perform well in high-quality post-mortem samples.

We compared the impact of RNA degradation on both long-read Nanopore DRS and PCR-cDNA as this has not been previously established. We found that PCR introduces amplification biases and that DRS provides a more accurate representation of the native molecules present within samples. DRS was more suitable for the identification and quantification of longer molecules even in the context of partially degraded samples. It is important to note that PCR-cDNA sequencing allows sample multiplexing, lower inputs and also produces ∼10× more reads. This results in increased detection of genes and isoforms, making PCR-cDNA more suited to experiments requiring higher throughput. Furthermore, the number of cDNA datasets we generated was limited, and further research is necessary to fully understand the impact of RNA degradation on this sequencing method.

### Recommendations regarding the inclusion of RNA samples for DRS

Previous studies assessing the impacts of RNA degradation on short-read sequencing have recommended a range of RIN values that can be used for RNA-seq (5,16,50). DRS is also sensitive to RNA quality and only samples with a RIN >9.5 can truly be considered undegraded. For DE analyses, we recommend using samples with a RIN > 9 as we observe few differences between gene and isoform measurements at this level, as evidenced by the low number of genes and isoforms identified as DE between RINs from these time points.

Obtaining such high-quality samples is not always feasible; however, our results suggest that useful results can still be obtained from DRS in samples with a RIN > 7 if appropriate correction steps are implemented. We highly recommend NanoCount filtering in conjunction with explicit RIN correction to mitigate confounders due to degradation. Although our study did not test DRS on samples below a RIN of 7, the increasingly significant and pervasive changes that degradation introduces means we would recommend approaching data derived from such samples with caution, though useable data are likely obtainable below this range.

In summary, we show that degradation induces widespread changes to the transcriptome and can have negative effects on DRS results. Nevertheless, explicitly accounting for sample quality is an effective approach for reducing the confounding effects of degradation and restoring meaningful biological signal. We conclude that DRS can be applied to samples with a range of RIN values from 7 to 10 and provide a framework to correct for the impact of degradation on DRS results.

## DATA AVAILABILITY

FAST5 and FASTQ files are publicly available to download from ENA: PRJEB53210. FASTQ files from the post-mortem brain sample are available at EGA: EGAS00001006542. All code used to perform the analysis is available on GitHub: https://github.com/Sefi196/RNA_Deg. BamSlam is available at https://github.com/josiegleeson/BamSlam. Permanent DOI: 10.5281/zenodo.7970821.

## Supplementary Material

lqad060_Supplemental_Files
