# Supplementary material for: Pervasive effects of RNA degradation on Nanopore direct RNA sequencing
Source: NAR Genom Bioinform. 2023 Jun 9;5(2):lqad060. doi: 10.1093/nargab/lqad060 (PMC10251640; doi:10.1093/nargab/lqad060)
Supplement: lqad060_Supplemental_Files [file lqad060_supplemental_files.zip › revised2_Supplementary_Figures_Combined.pdf]

Figure S1

All isoforms

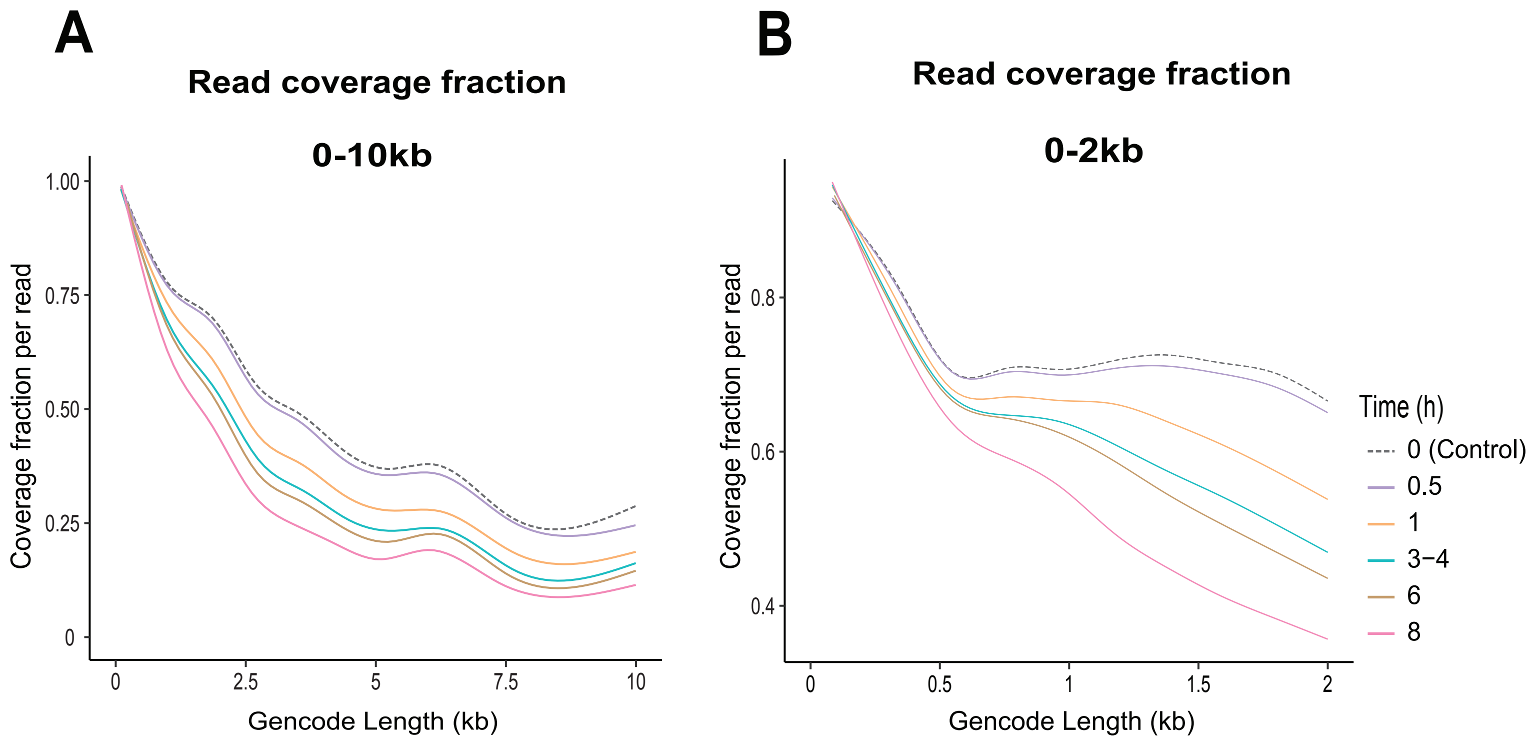

Single isoform genes

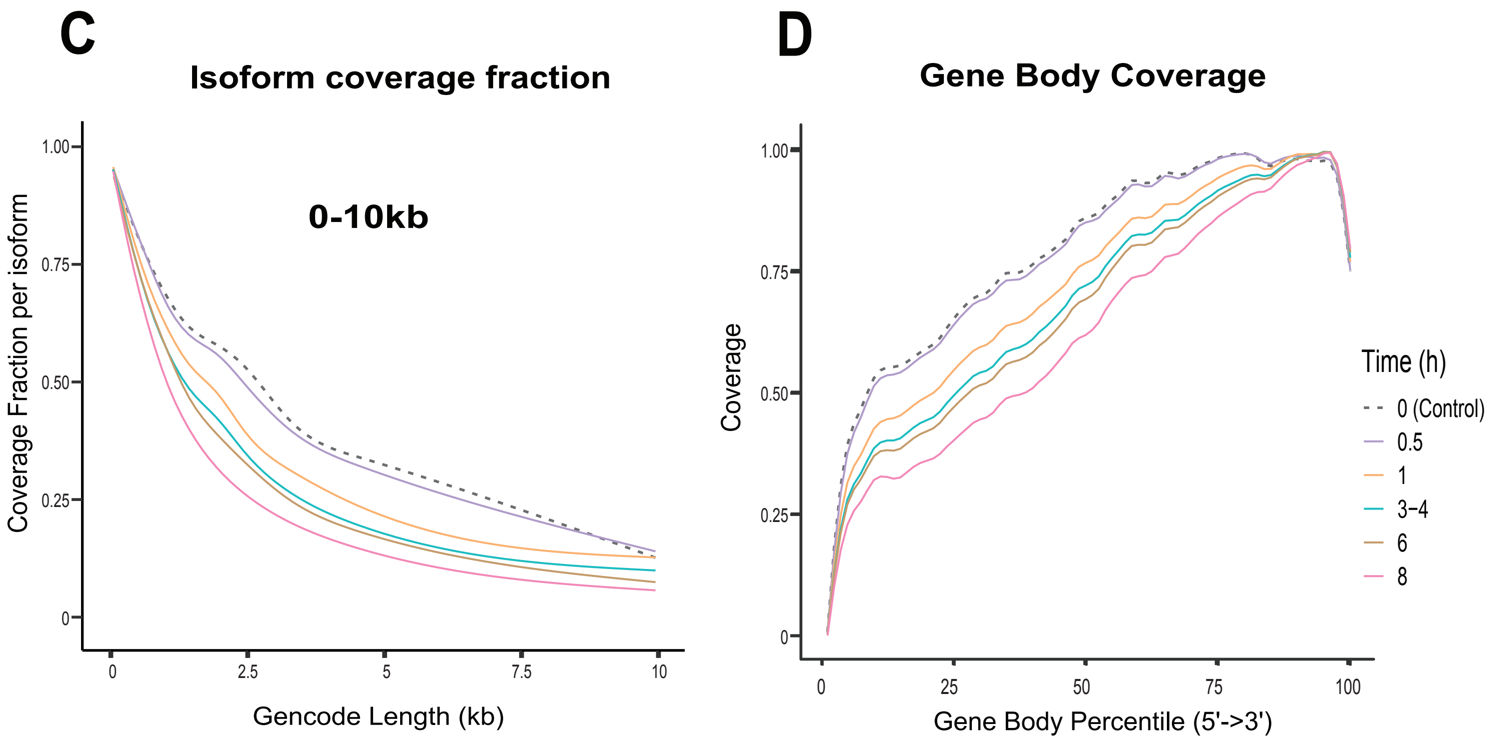

Figure S1. Coverage fractions. (A) Coverage fractions ranging from 0 to 10kb and **(B)** 0 to 2kb for all reads mapped to an isoform compared with known isoform length. (**C**) Isoform coverage fractions for single isoform genes ranging from 0 to 10 kb. (**D**) Gene body coverage for single isoform genes. Length of all genes normalised to 100 and plotted from 5' (0) to 3' (100). Lines show mean coverage for all genes across the gene body length. Lower coverage at extreme 3' corresponds to soft clipping of the first bases sequenced which often have lower phred quality.

Figure S2

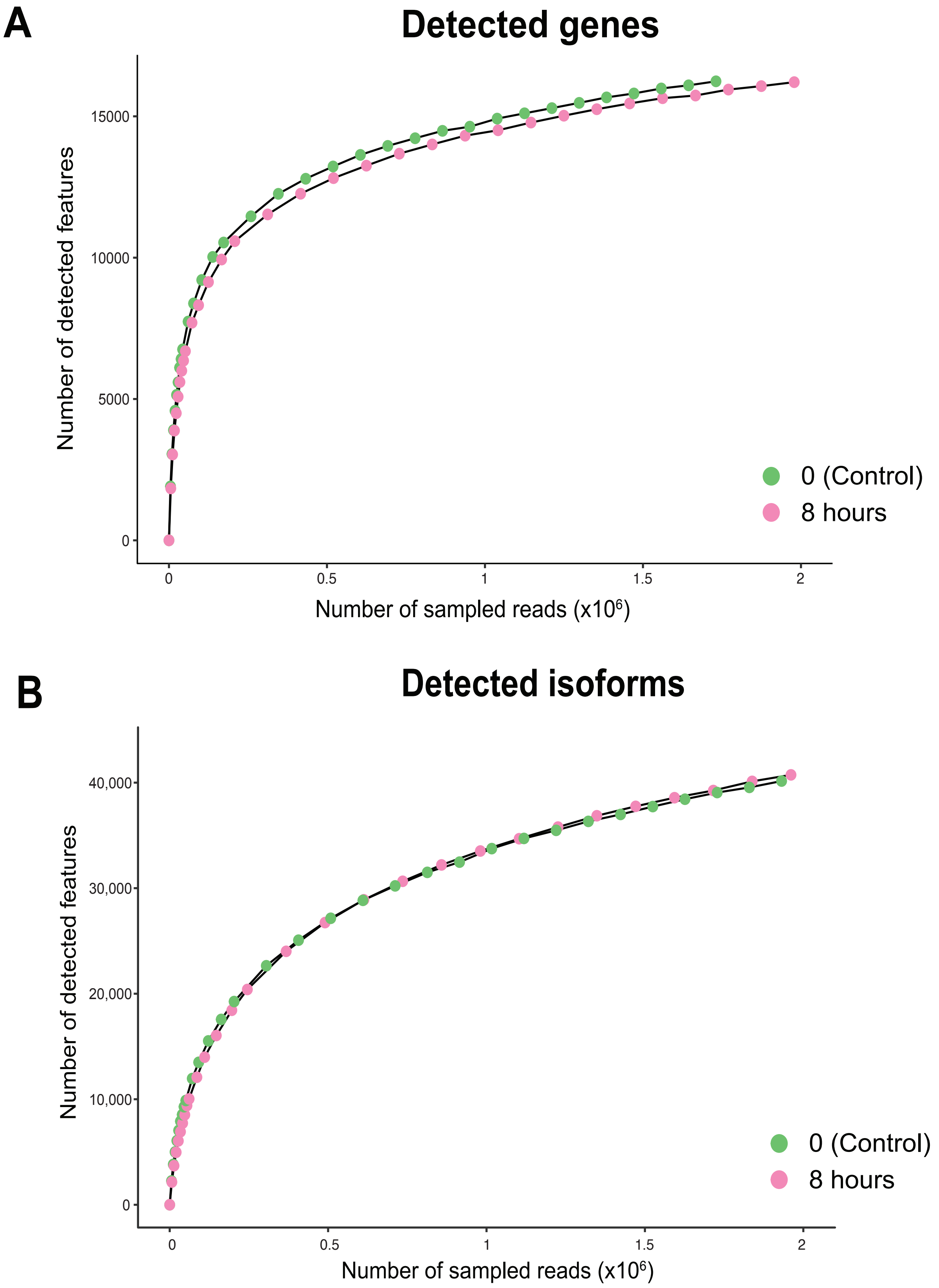

Figure S2. Gene and isoform rarefaction curves. (A) Number of detected genes **(B)** and isoforms detected in the 0-hour control and 8-hour samples.

Figure S3

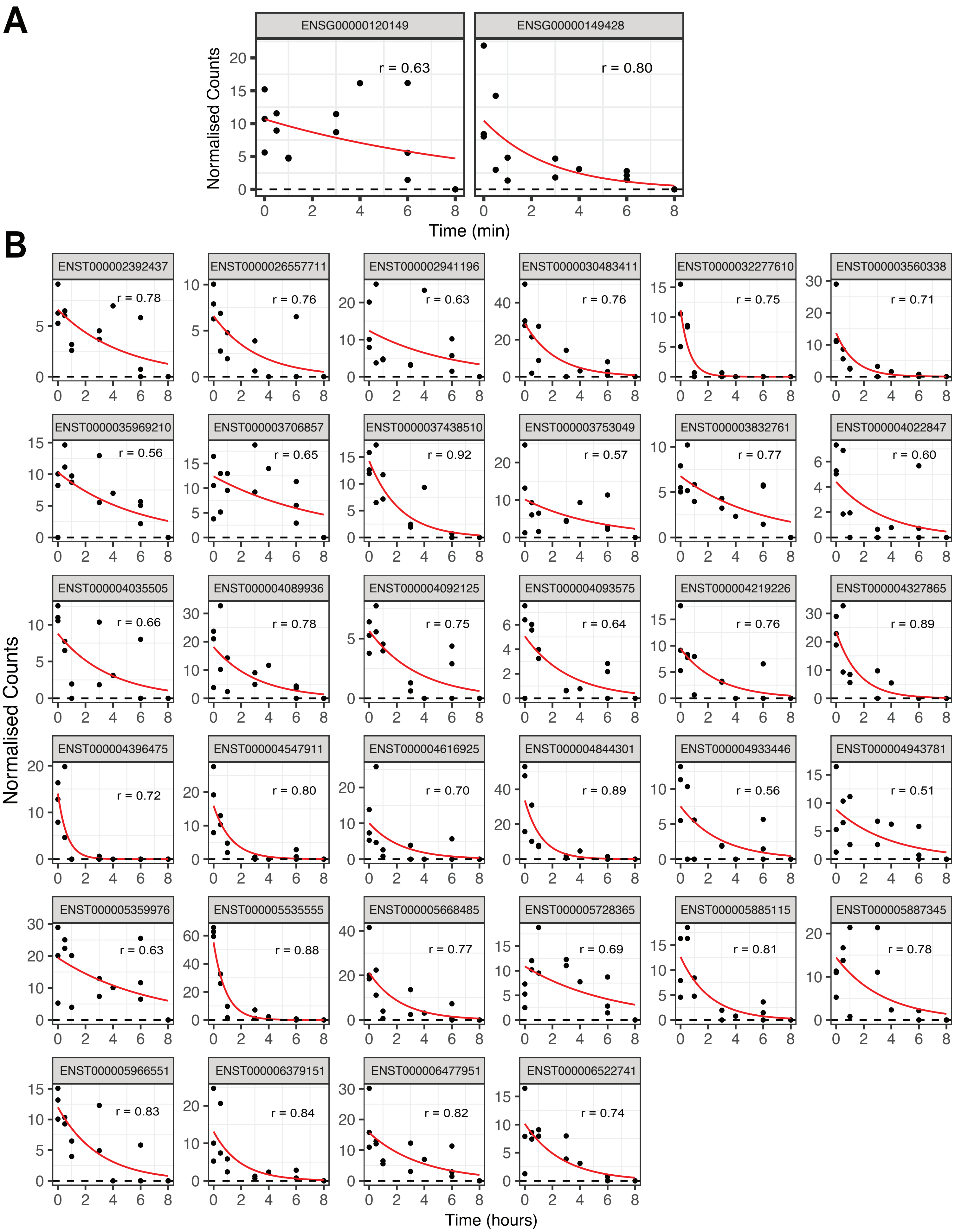

Figure S3. Genes and isoforms that decay to non-detectable levels by 8 hours. (A) genes and **(B)** isoforms with zero counts at 8 hours. Red lines are exponential decay curves. r = Pearson's correlation coefficient.

Figure S4

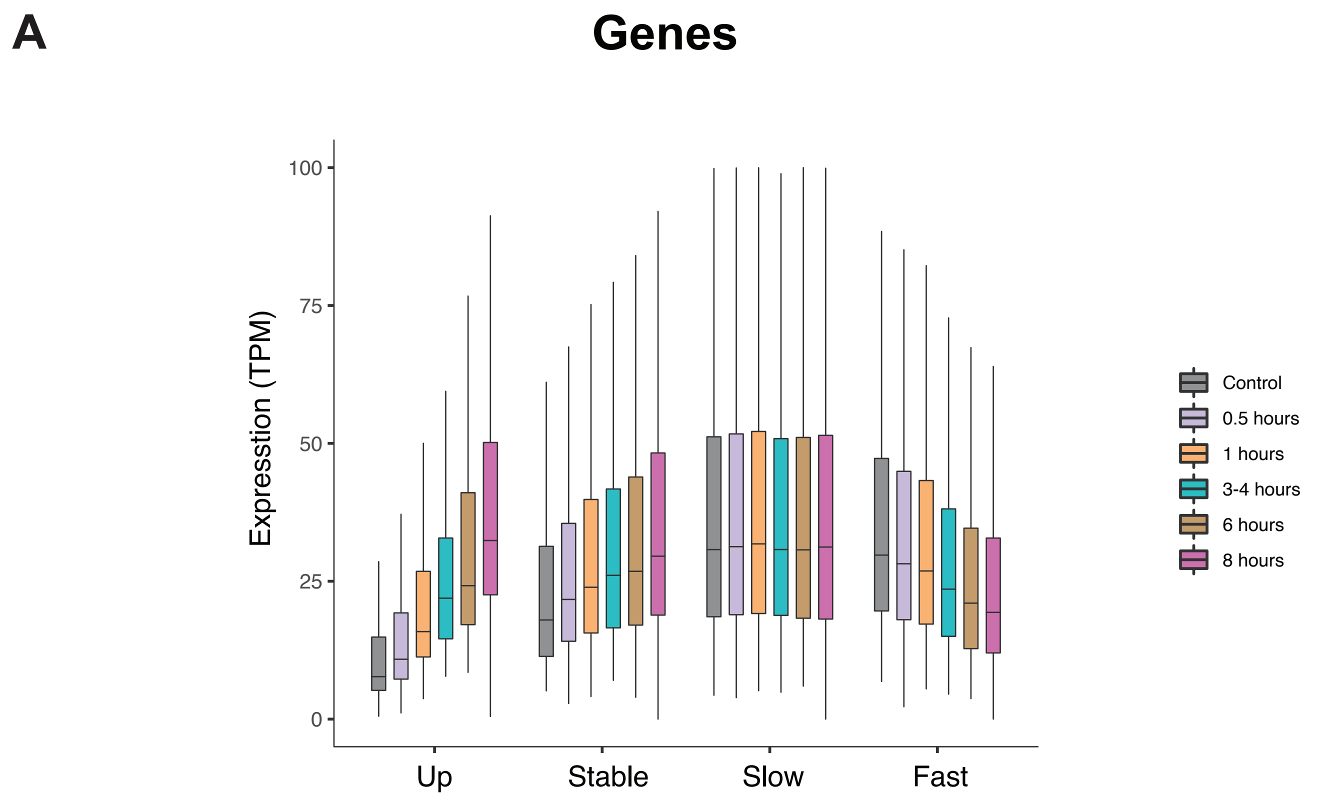

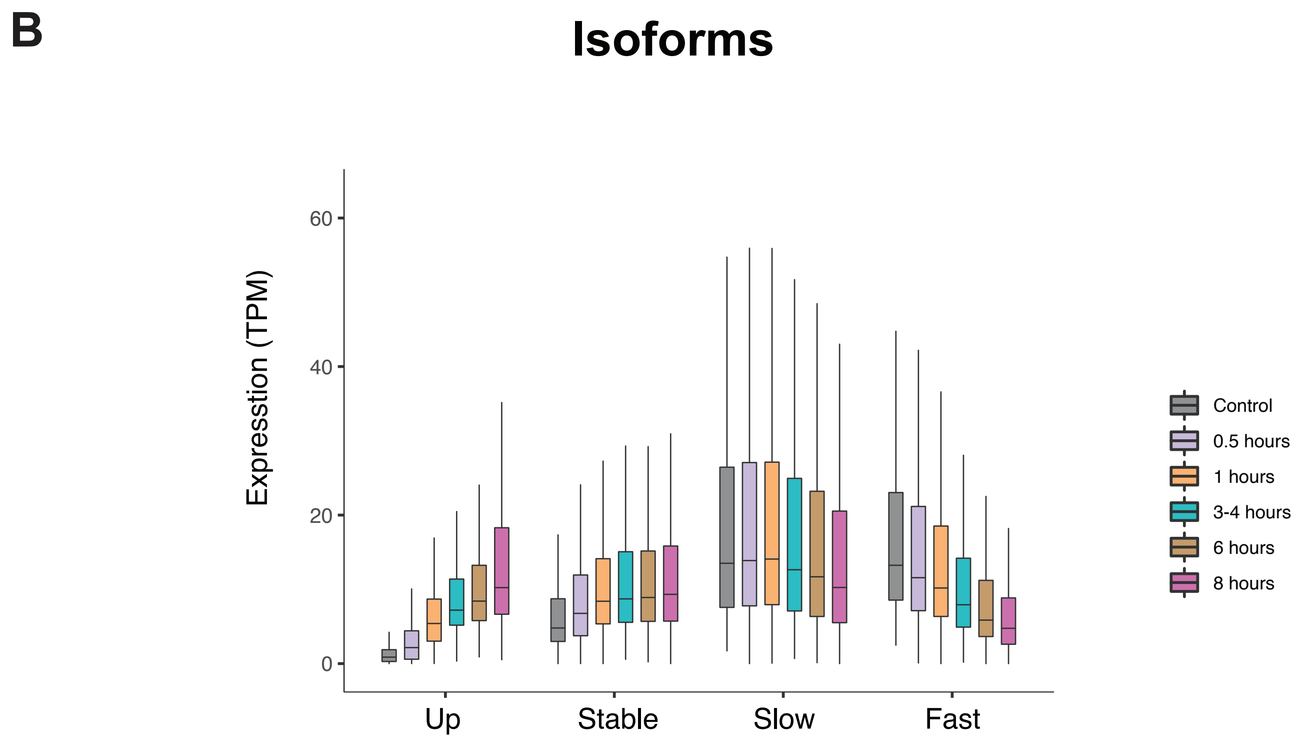

Figure S4. Association between expression levels and degradation clusters. Up = upregulated, slow = slow degradation, fast = rapid degradation. **(A)** Genes and **(B)** isoform expression values in TPM. In the interest of clarity panels omit outliers.

Figure S5

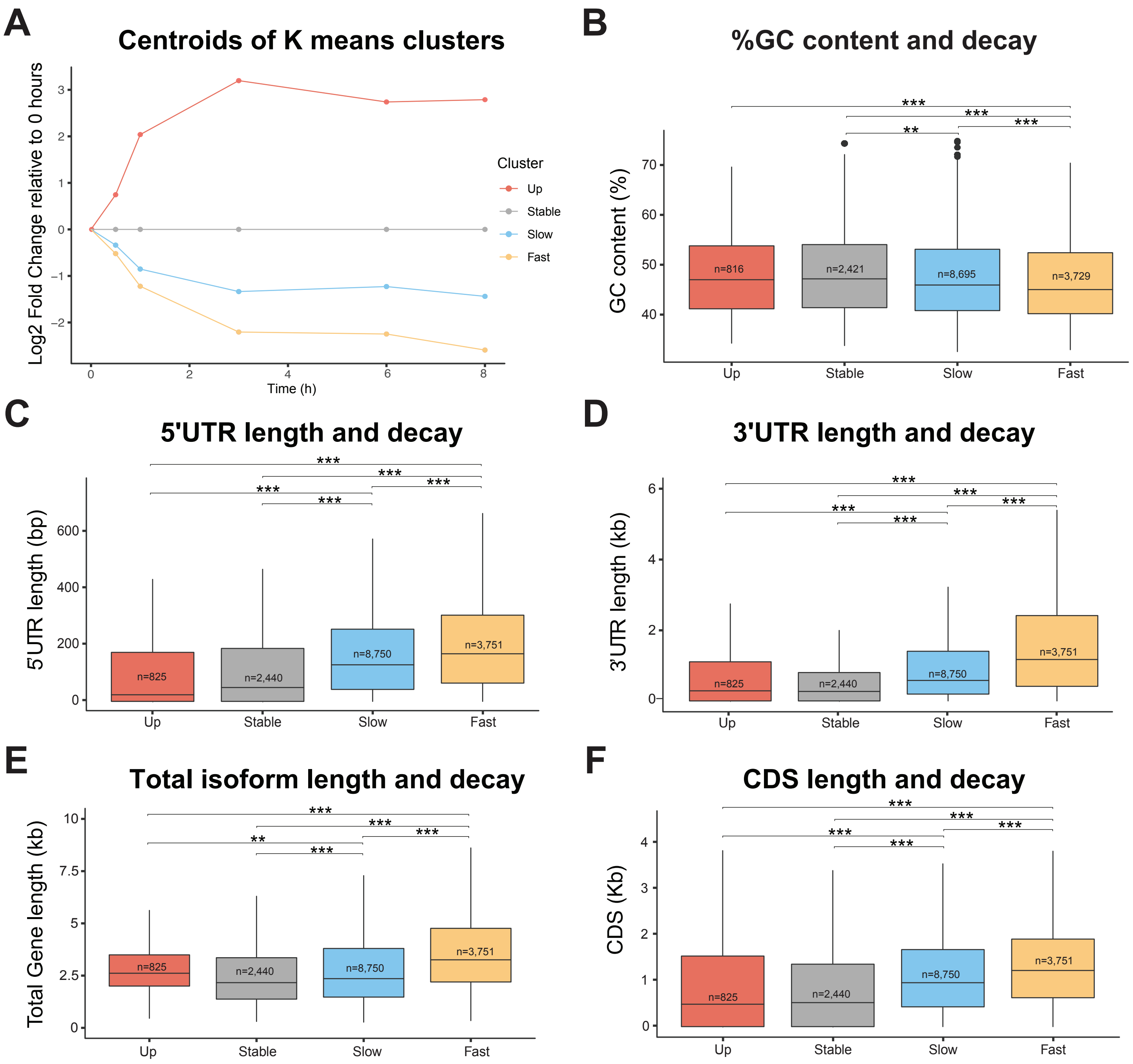

Figure S5. Characteristics of isoform degradation clusters. Up = upregulated, slow = slow degradation, fast = rapid degradation. **(A)** Centroid of each K means cluster derived from log2 fold change data scaled to 0 hours. **(B-F)** GC% **(B),** 5' UTR length **(C),** 3'UTR length **(D),** complete transcript length (UTRs + CDS) **(E)** and CDS length **(F)** of isoforms in each cluster. In the interest of clarity panels C-F omit outliers.Statistical comparisons between clusters were performed with an ANOVA and post-hoc analysis using Tukey's test. Statistical comparisons between clusters were performed with an ANOVA and post-hoc analysis using Tukey's test and can be found in Additional file 3. Outliers are included in the statistical analysis. P-values: * < 0.05, ** < 0.01, *** < 0.001.

Figure S6

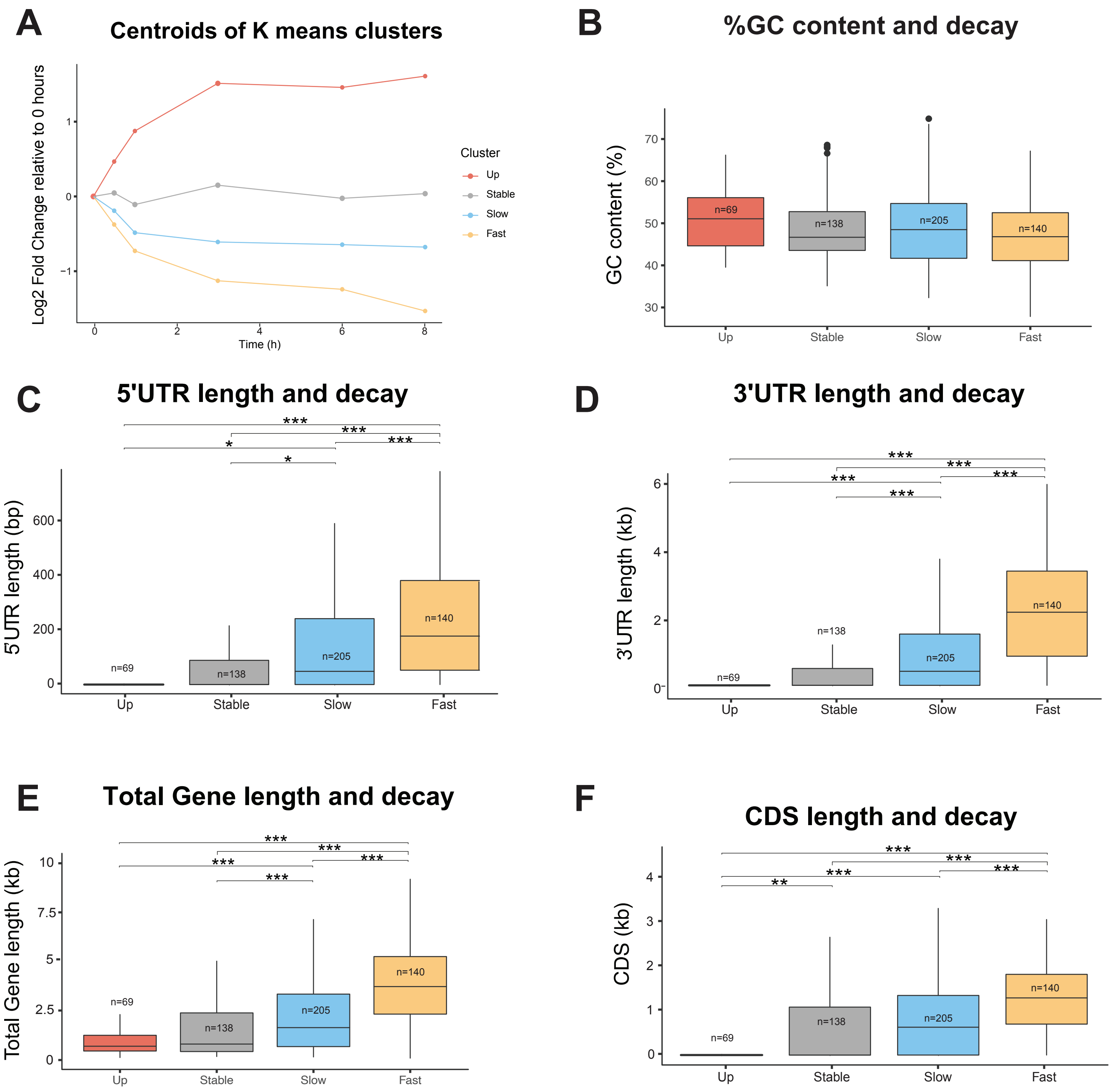

Figure S6. Characteristics of isoform degradation clusters for single isoform genes. Up = upregulated, slow = slow degradation, fast = rapid degradation. **(A)** Centroid of each K means cluster derived from log2 fold change data scaled to 0 hours. **(B-F)** GC% **(B),** 5' UTR length **(C),** 3'UTR length **(D),** complete transcript length (UTRs + CDS) **(E)** and CDS length **(F)** of isoforms in each cluster. In the interest of clarity panels C-F omit outliers. Statistical comparisons between clusters were performed with an ANOVA and post-hoc analysis using Tukey's test. Outliers are included in the statistical analysis. P-values: * < 0.05, ** < 0.01, *** < 0.001.

Figure S7

A NanoCount

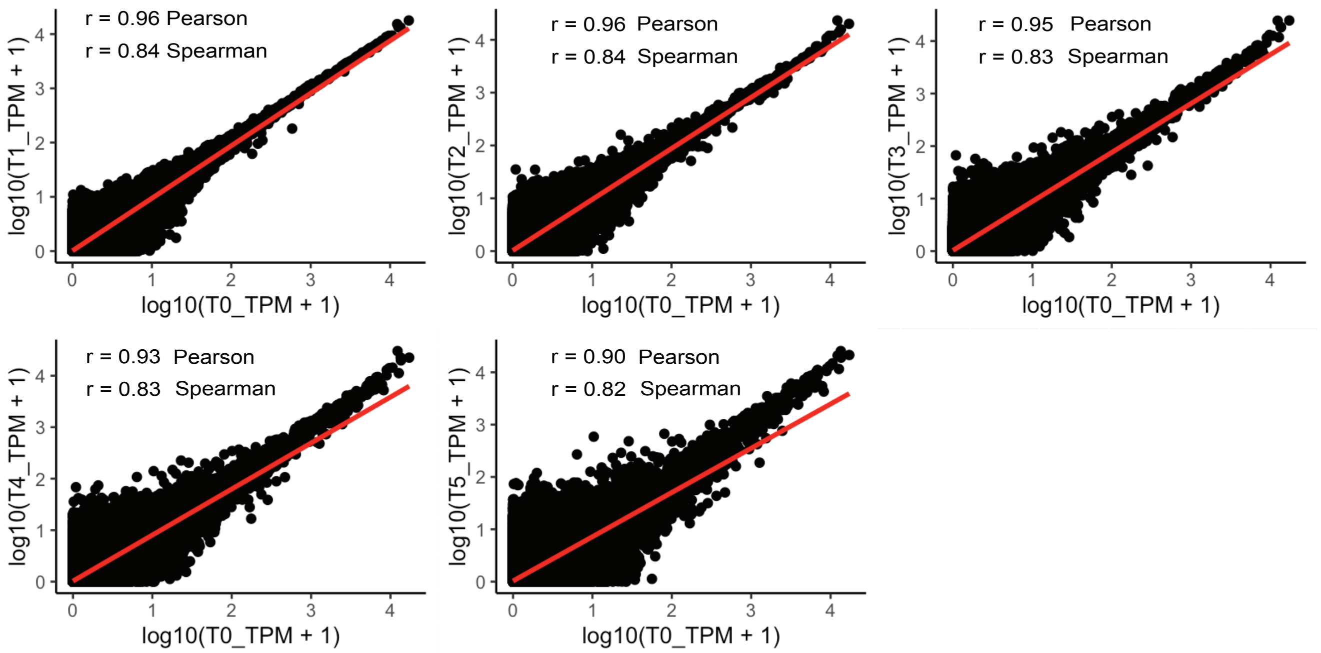

B Salmon

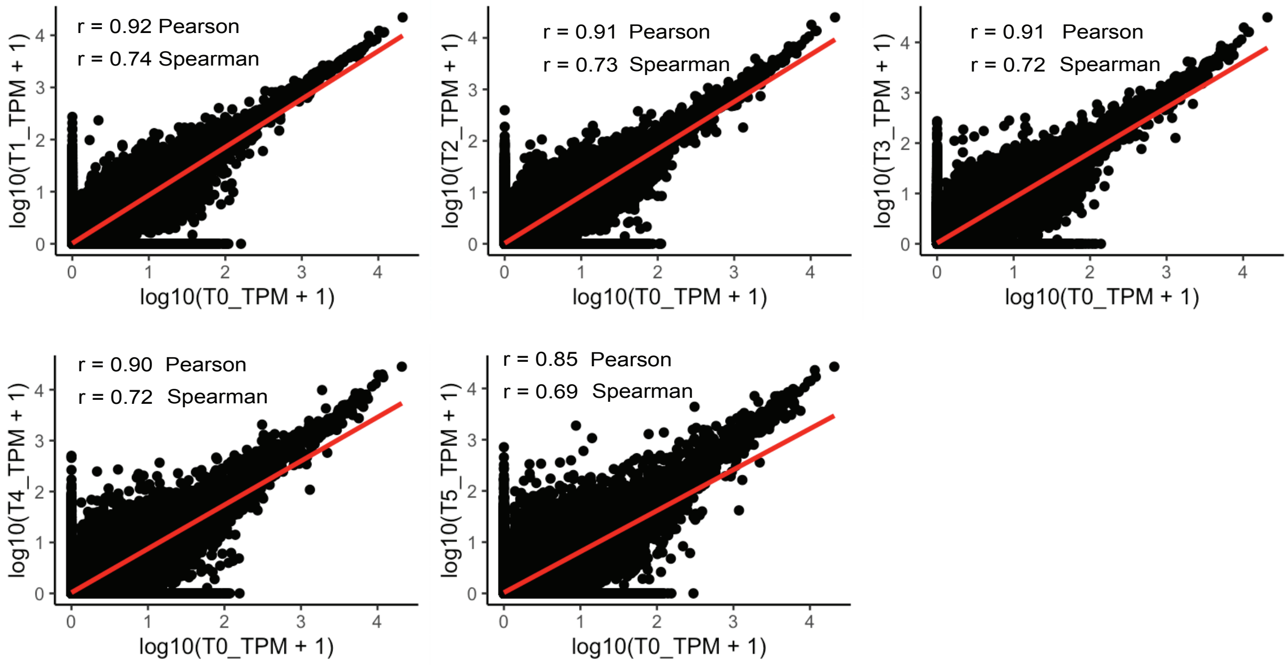

Figure S7. Correlation of TPM values at each time point compared to control (T0). A) NanoCount and **B)** Salmon. Data is plotted on log10 scale and both Pearson's and Spearman's correlation coefficients are reported. A linear regression has been fit to each plot and is shown in red.

Figure S8

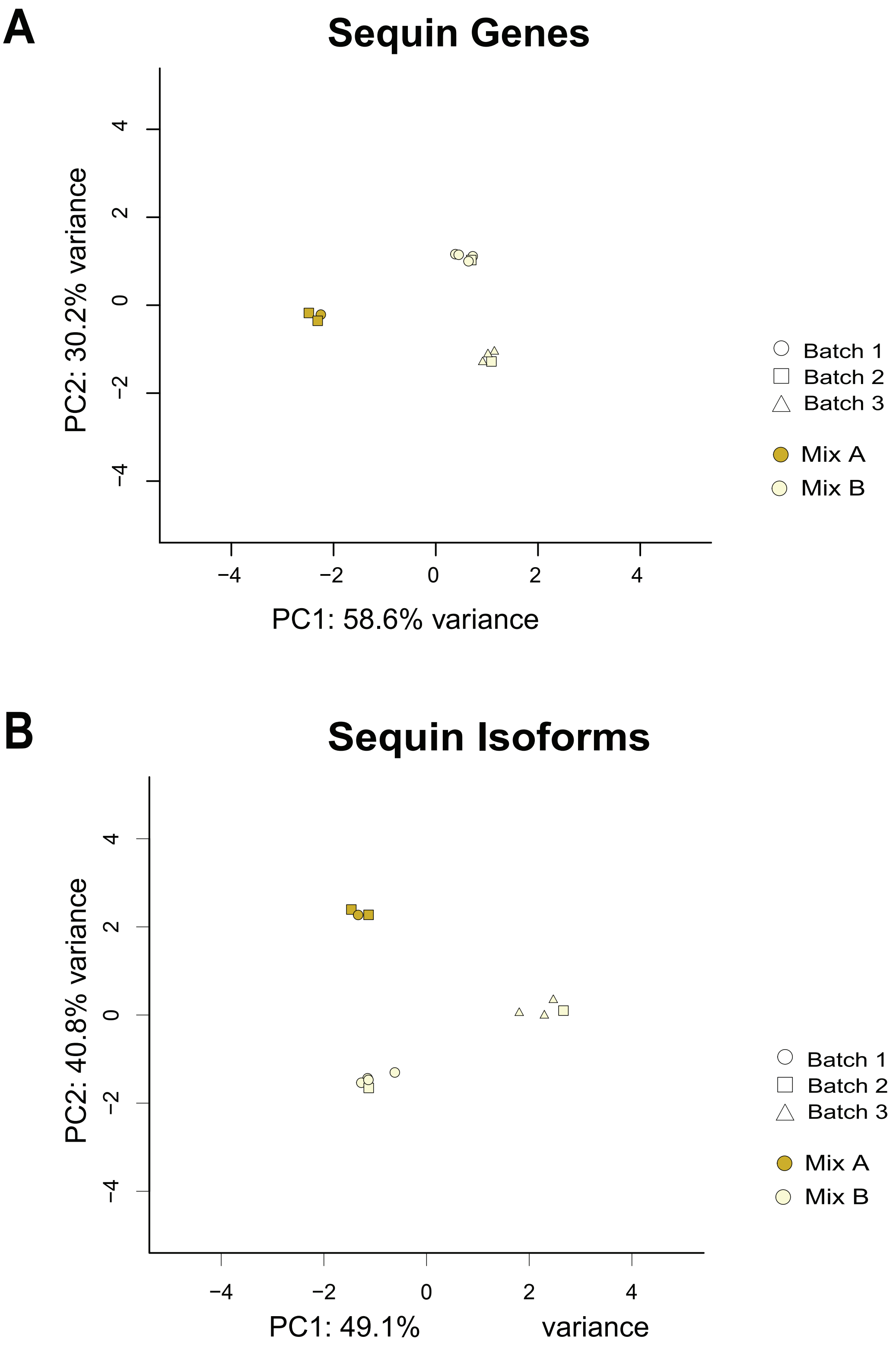

Figure S8. Sequin PCA. PCAs generated with **(A)** sequin gene and **(B)** transcript counts from FeatureCounts and NanoCount respectively. Samples separate by sequin mix along PC1 for genes (58.6% of the variance) and PC2 for isoforms (40.8% of the variance). A batch effect was observed as mix B samples separate into two groups, likely the result of a freeze-thaw cycle in the normal process of sequin handling and storage affecting sequin stability. To mitigate possible confounding effects, sequin batch was included in the GLM (see Methods).

Figure S9

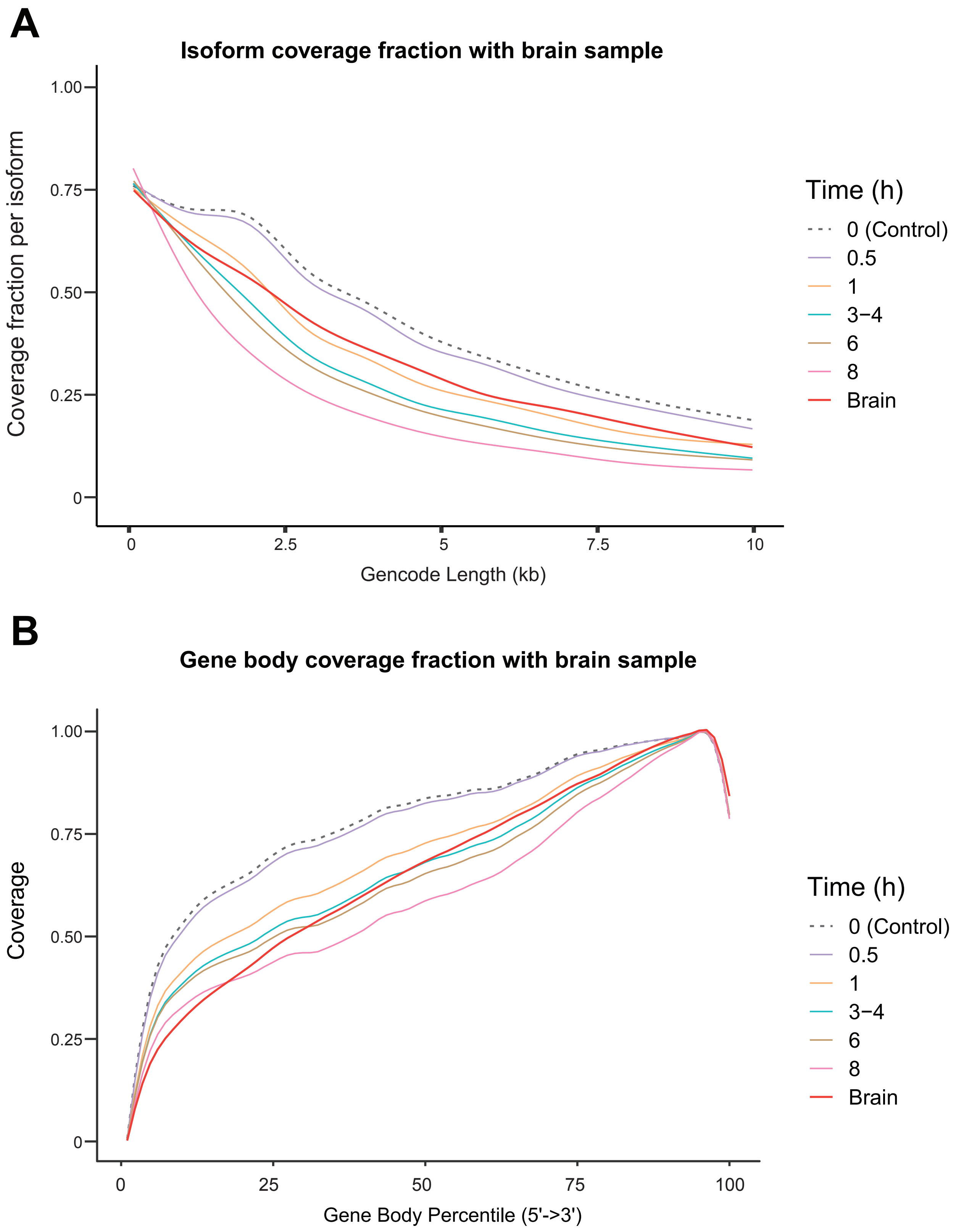

Figure S9. Coverage fractions with inclusion of the cerebellum brain sample. (A) Isoform coverage fractions ranging from 0 to 10 kb. Median coverage fraction for all reads mapped to an isoform compared to the known isoform length. The cerebellum brain sample coverage fraction is shown in bold red. **(B)** Gene body coverage with inclusion of the cerebellum brain sample. Length of all genes normalised to 100 and plotted from 5' (0) to 3' (100). Lines show mean coverage for all genes across the gene body length. Lower coverage at extreme 3' corresponds to soft clipping of the first bases sequenced which often have lower phred quality. Cerebellum brain sample shown in bold red.
