# Supplementary material for: Pervasive effects of RNA degradation on Nanopore direct RNA sequencing
Source: NAR Genom Bioinform. 2023 Jun 9;5(2):lqad060. doi: 10.1093/nargab/lqad060 (PMC10251640; doi:10.1093/nargab/lqad060)
Supplement: lqad060_Supplemental_Files [file lqad060_supplemental_files.zip › Supplementary table legends.docx]

**Supplementary table legends**

Table S1: Time series summary statistics. Table containing summary statics and QC metrics for all samples in the manuscript including information on sequins.

Table S2: Direct RNA sequencing metrics filtered with NanoCount. Table containing sequencing metrics post NanoCount filtering.

Table S3: Statistical tests for gene architecture analysis. Supplementary table showing the output from the statistical tests that were performed. The three tests performed were an ANOVA, Eta squared (h^2^) to measure effect size and Tukey HSD post hoc test for significant pairwise comparisons.

Table S4: GO analysis for each degradation cluster. GO results for each degradation cluster with associated GO terms, ontology and significance value.

Table S5: Molecular chaperones in the upregulated cluster. Table showing the 10 molecular chaperones found in the upregulated cluster including their protein family and class.
